# Comprehensive characterization of respiratory genes based on a computational framework in pan-cancer to develop stratified treatment strategies

**DOI:** 10.1371/journal.pcbi.1012963

**Published:** 2025-04-09

**Authors:** Caiyu Zhang, Chenyu Liu, Zhuoru Wang, Di Wang, Wenli Chen, Jian Li, Qianyi Lu, Jiajun Zhou, Yetong Chang, Peng Wang, Yue Gao, Junwei Wang, Hui Zhi, Shangwei Ning

**Affiliations:** 1 College of Bioinformatics Science and Technology, Harbin Medical University, Harbin, Heilongjiang, China; 2 Department of Respiratory Medicine, The Second Affiliated Hospital of Harbin Medical University, Harbin, China; Yale University, UNITED STATES OF AMERICA

## Abstract

Abnormal cellular respiration plays a critical role in carcinogenesis. However, the molecular mechanisms underlying dysregulation of respiratory gene expression across different cancer types remain unclear. Here, we developed a computational framework that provides an analytical approach for exploring the molecular alterations and clinical relevance of respiratory genes in pan-cancer. We identified a total of 53 gene signatures in the three stages of respiration (including glycolysis, tricarboxylic acid cycle, and oxidative phosphorylation) through this framework and found that they were broadly differentially expressed and genetically altered across 33 cancer types. Pathway analysis manifested that the expression levels of almost all respiratory gene signatures were remarkably associated with the activation or inhibition of numerous oncogenic pathways, such as metabolism, angiogenesis, cell proliferation, and apoptosis. Survival analysis highlighted the oncogenic or tumor suppressor potential of the respiratory gene signatures. In particular, *VCAN* has shown significant oncogenic features in multiple cancer types. Finally, we identified a number of respiratory gene signatures that could be potential therapeutic targets, including *VCAN*. We also predicted small-molecule compounds targeting respiratory gene signatures or components of pathways regulated by them. Overall, our comprehensive analysis has greatly enhanced the understanding of molecular alterations of respiratory genes in tumorigenesis and progression, and provided insights into developing new therapeutic strategies.

## Author summary

A critical factor in carcinogenesis is the dysregulation of cellular respiration. In this study, we explored the molecular alterations and clinical relevance of respiratory genes in pan-cancer and developed stratified treatment strategies for specific patients. We conducted a two-step computational framework to identify gene signatures in the three stages of respiration (including glycolysis, tricarboxylic acid cycle, and oxidative phosphorylation). By analyzing data from different TCGA cancer types, we found that respiratory genes had aberrant expression patterns and extensive genetic alterations across cancers. Moreover, we observed a significant correlation between the expression of respiratory gene signatures and the activation or inhibition of oncogenic pathways. Survival analysis demonstrated that the alteration of respiratory gene expression has a significant impact on patient outcomes. High expression of *VCAN* was associated with poor prognosis of patients in multiple cancers. Finally, we screened several respiratory gene signatures that could be potential therapeutic targets, such as *VCAN*, and predicted small-molecule compounds that target gene signatures.

## Introduction

A key factor in carcinogenesis is the dysregulation of cellular respiration [1]. The unique metabolic feature of tumor cells, the transition between respiration and fermentation, can rapidly provide energy and promote their growth and proliferation [2,3]. Therefore, the expression perturbation of respiratory genes may be closely related to cancer progression [4]. Over the past few years, a great deal of work has been done to elucidate the altered energy metabolism of cancer cells, which adapt to environmental challenges through enhanced glycolysis [5–7]. Nevertheless, most studies have not performed a comprehensive and multidimensional characterization of the molecular mechanisms underlying respiratory gene dysregulation in a broad range of cancer types.

Cellular respiratory metabolism is divided into three stages: glycolysis, the tricarboxylic acid (TCA) cycle, and oxidative phosphorylation [8]. Glycolysis is an indispensable part of malignant tumorigenesis [9]. The study by Jinshou Yang et al. showed enhanced glycolysis in pancreatic cancer cells and revealed a close relationship between glycolysis and malignant transformation of cells [10]. As a metabolic hub, the TCA cycle is a central pathway connecting almost all metabolic pathways in organisms [11]. In parallel, intermediates of the TCA cycle have also been shown to promote or inhibit cancer progression [12]. Oxidative phosphorylation is the final process of cellular respiration and an essential energy factory [13]. Reports have shown that certain cancer types could also suppress the expression of genes involved in the oxidative phosphorylation stage [3]. However, our current knowledge of the molecular mechanisms by which these three stages are involved in tumorigenesis remains limited.

As the understanding of the respiration process in tumor cells has deepened, its complex biological function has also begun to be widely explored. The close link between cellular respiration and signaling pathways might facilitate a wide range of clinical applications [14]. Evidence showed that inhibiting NF-κB pathway could improve glycolysis, thereby promoting hematopoietic stem/progenitor cells (HSPCs) ex vivo expansion [15]. Altered expression of WNT/β-catenin signaling pathway could regulate aerobic glycolysis and the progression of amyotrophic lateral sclerosis [16]. These findings indirectly highlighted the potential of respiratory genes in cancer treatment and provided new ideas for developing anti-cancer approaches with targeted therapy [17]. However, previous studies lacked precise delineation of patients with dysregulated respiratory processes and were thus inadequate for stratified treatment [18].

To address the above issues, we first identified 53 respiratory gene signatures and performed a comprehensive analysis of genetic alterations and differential expression of respiratory gene signatures across 33 cancer types. Subsequently, we preliminarily identified several cancer hallmark-related pathways regulated by respiratory gene signatures and further validated them through enrichment analysis. Given that the respiratory gene signatures of distinct stages may not function independently in cancer progression, we also observed co-expression patterns (including gene- and protein-level) among the three stages of respiration. Next, we assessed the clinical relevance of respiratory gene signatures in pan-cancer and identified an oncogene, *VCAN*, whose high expression was associated with poor prognosis in multiple cancers. Finally, we predicted respiratory gene signatures that could be potential therapeutic targets. We have also developed candidate small-molecule compounds that target respiratory gene signatures or oncogenic pathways regulated by them for patients with specific stage dysregulation. To the best of our knowledge, this is a systematic study of a multidimensional molecular portrait of respiratory genes across various cancer types at an unprecedented level.

## Materials and methods

### Genome-wide omics data across 33 cancer types

The results of our analysis are based on the datasets of multi-omics and clinically relevant features derived from TCGA Research Network (https://cancergenome.nih.gov/). In total, 33 various cancer types were analyzed, each with an acronym in accordance with the TCGA nomenclature. The details were as follows: adrenocortical carcinoma (ACC), bladder urothelial carcinoma (BLCA), breast invasive carcinoma (BRCA), cervical squamous cell carcinoma and endocervical adenocarcinoma (CESC), cholangiocarcinoma (CHOL), colon adenocarcinoma (COAD), lymphoid neoplasm diffuse large B-cell lymphoma (DLBC), esophageal carcinoma (ESCA), glioblastoma multiforme (GBM), head and neck squamous cell carcinoma (HNSC), kidney chromophobe (KICH), kidney renal clear cell carcinoma (KIRC), kidney renal papillary cell carcinoma (KIRP), acute myeloid leukemia (LAML), brain low grade glioma (LGG), liver hepatocellular carcinoma (LIHC), lung adenocarcinoma (LUAD), lung squamous cell carcinoma (LUSC), mesothelioma (MESO), ovarian serous cystadenocarcinoma (OV), pancreatic adenocarcinoma (PAAD), pheochromocytoma and paraganglioma (PCPG), prostate adenocarcinoma (PRAD), rectum adenocarcinoma (READ), sarcoma (SARC), skin cutaneous melanoma (SKCM), stomach adenocarcinoma (STAD), testicular germ cell tumors (TGCT), thyroid cancer (THCA), thymoma (THYM), uterine corpus endometrial carcinoma (UCEC), uterine carcinosarcoma (UCS), uveal melanoma (UVM). All data, including gene expression, methylation, mutations, and copy number, were downloaded from the Xena Browser (https://xenabrowser.net/datapages/).

### The collection of respiratory genes

We manually collected and collated 661 genes from the Molecular Signatures Database (MsigDB) (http://www.gsea-msigdb.org/gsea/msigdb/) and Kyoto Encyclopedia of Genes and Genomes (KEGG, RRID:SCR_012773) (https://www.genome.jp/kegg/) by retrieving the three stages of respiration, namely glycolysis, tricarboxylic acid cycle, and oxidative phosphorylation. These included 304 glycolysis-, 57 tricarboxylic acid cycle- and 300 oxidative phosphorylation-related genes.

### The computational framework for identifying respiratory gene signatures

We performed a two-step analysis to identify respiratory gene signatures of three stages. First, we screened respiratory genes significantly differentially expressed in 17 cancer types with at least five normal samples. Next, we conducted single sample gene set enrichment analysis (ssGSEA) with the three stages of respiration as functional pathways. We further divided samples of three stages into high and low groups based on the median values of enrichment scores and selected significantly differentially expressed genes. Finally, the respiratory gene signatures were identified through both analyses above.

### Differential expression analysis of respiratory gene signatures

First of all, we selected 17 cancer types with at least five normal samples. A t-test was then used to identify gene signatures that were markedly differentially expressed between normal and tumor tissue across 17 cancers, and p-values were adjusted using the BH method. Gene signatures with FDR values less than 0.05 and absolute values of fold change greater than 1 were considered to be significantly differentially expressed.

### Differential methylation analysis of respiratory gene signatures

Differential methylation analysis between cancer and normal samples was conducted using R package ‘limma’ and p-values were adjusted using the Benjamini-Hochberg (BH) method. Gene signatures with a log fold change higher than 0.1 and a p-value less than 0.05 were considered significantly differentially methylated.

### The identification of cancer hallmark-related pathways regulated by respiratory gene signatures

As a first step, we conducted a single sample gene set enrichment analysis (ssGSEA) on the normalized gene expression values to calculate the activity of 50 cancer hallmark-related pathways [19]. Next, we calculated Pearson correlation coefficients between oncogenic pathway activity and the expression of 53 respiratory gene signatures. Cancer hallmark-related pathways with p-values less than 0.01 and absolute values of Pearson correlation coefficients greater than 0.6 were considered to be notably regulated by respiratory gene signatures.

### Correlation patterns between respiratory gene signatures in the diverse stages

We calculated Pearson correlation coefficients of respiratory gene signatures with each other in the three stages based on gene expression among 33 cancer types. Additionally, we also identified the protein-protein interaction network of respiratory gene signatures across different stages utilizing the Retrieval of Interacting Genes Database (STRING, RRID:SCR_005223) (https://www.string-db.org/) [20]. The results were displayed through Cytoscape (RRID:SCR_003032), an open source platform for network visualization and data integration [21].

### Survival analysis

All clinical information of patients was downloaded from the publicly available TCGA project (https://portal.gdc.cancer.gov/) and the GEO database (https://www.ncbi.nlm.nih.gov/geo/). We compared the overall survival between two groups of patients divided according to the median value of the expression of respiratory gene signature. Kaplan-Meier survival curves were exerted to visualize the prognostic differences between two groups of patients. P-values were calculated by log-rank test, and those less than 0.05 were thought to be significant.

### Gene set enrichment analysis

At first, we divided samples in the three kidney cancer subtypes into high and low expression groups, respectively, based on the median expression value of the oncogene *VCAN*. After that, we calculated and ranked the fold-change values of all genes in the two sets of samples. Finally, we conducted Gene Set Enrichment Analysis (GSEA, RRID:SCR_003199) on the pre-ranked gene list using the R package clusterProfiler (RRID:SCR_016884). The gene sets c5.go.v7.4.symbols, c2.cp.kegg.v7.4.symbols, and h.all.v7.4.symbols were downloaded from the MsigDB database. Adjusted p-values less than 0.05 were considered statistically significant.

### Calculating the glycolysis and oxidative phosphorylation score

We first Z-normalized the expression values of glycolysis- and oxidative phosphorylation-related gene signatures within each cancer type. The glycolysis and oxidative phosphorylation scores per sample were defined as the mean of normalized expression values of the glycolysis- and oxidative phosphorylation-related gene signatures in each sample, respectively. The score represents the overall estimated level of respiratory gene signatures in these two stages [22].

### Identification of potential drug targets

After obtaining the glycolysis and oxidative phosphorylation scores for each sample, we calculated Pearson correlation between the expression levels of drug targets and sample scores. Drug targets with a correlation coefficient greater than 0.3 and a p-value less than 0.05 were selected and intersected with the 53 gene signatures to identify respiratory genes that could serve as potential drug targets.

### Identification of candidate small molecule compounds by Connective Map analysis

Within each cancer type, Connectivity Map analysis (CMap) (http://clue.io/) was carried out on the top 150 genes that were positively and negatively correlated with each sample’s glycolysis and oxidative phosphorylation scores. Small-molecule compounds with enrichment scores greater than 95 or less than -95 were considered to have potential therapeutic effects [22].

## Results

### Respiratory gene signatures are widely differentially expressed and genetically altered across various cancer types

**Fig 1:**
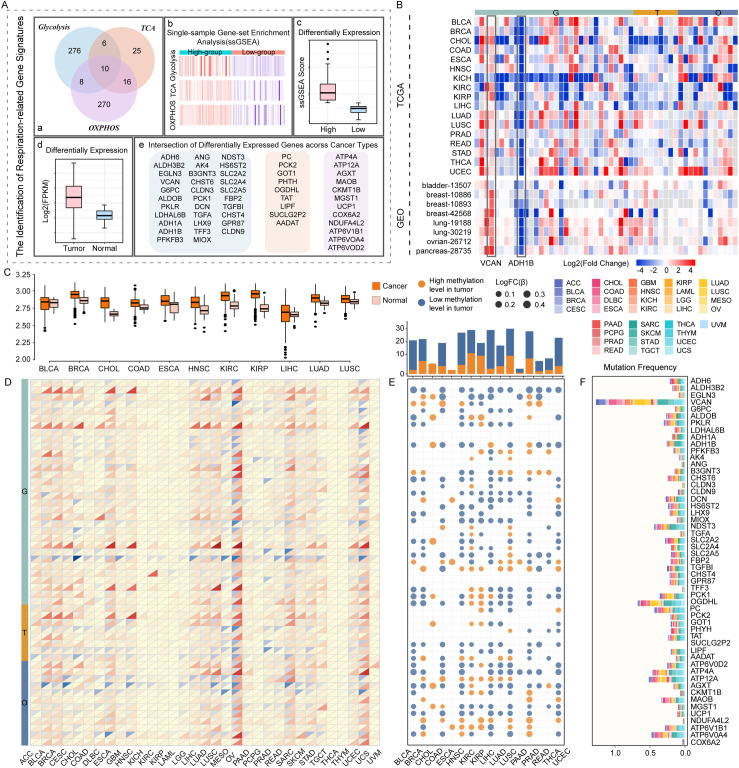
Differential expression and genetic alterations of respiratory gene signatures in pan-cancer. (A) Summary of the process for identifying respiratory gene signatures in the three stages. (B) Heatmap shows the differential expression levels of respiratory gene signatures across various cancer types; up- and down-regulated gene signatures in tumor samples are marked in red and blue, respectively. (C) Box plots show the expression pattern of *VCAN* among tumor and normal samples in 11 cancer types. (D) The frequency of copy number amplification or deletion of respiratory gene signatures across cancer types. The upper triangle in red represents the amplification frequency, and the lower triangle in blue represents the deletion frequency. (E) The differential methylation levels of respiratory gene signatures across cancer types. Histogram (upper panel) shows the number of significantly hypermethylated and hypomethylated gene signatures in each cancer type, marked in orange and blue, respectively. Heatmap (bottom panel) shows the fold change in methylation values of respiratory gene signatures in tumor and normal samples. (F) Stacked column chart shows the mutation frequency of respiratory gene signatures across 33 cancer types.

We conducted a two-step framework to identify respiratory gene signatures of three stages. Differential expression analysis was performed between cancer and normal samples to select genes with significant differences. We then employed ssGSEA to calculate the activity levels of each stage in cellular respiration and identified differentially expressed genes between high- and low-score groups. Ultimately, the intersection of these two parts of genes was kept. Collectively, a total of 53 respiratory gene signatures were identified through this computational framework in 17 cancer types with sufficient normal samples, including 31 glycolysis-, 9 tricarboxylic acid cycle- and 13 oxidative phosphorylation-related gene signatures (Fig 1A). Notably, we classified gene signatures that intersected at different stages (*PC*, *PCK1*, *PCK2*, *GOT1*, and *PHYH*) according to their importance to each stage. For example, we identified *GOT1* in the GeneCards database as playing a major role in the TCA cycle and included it as a TCA cycle-related gene signature for subsequent analysis [23]. Next, we carried out differential expression analysis on tumor and normal samples of each cancer type to assess the altered expression patterns of respiratory gene signatures in different cancer contexts and subsequently validated them in the GEO data sets. The distribution of expression levels of respiratory gene signatures in these 17 TCGA cancer types and 8 GEO samples was displayed in Fig 1B. Out of the three stages of respiratory gene signatures, we noticed that four glycolysis-related gene signatures (e.g., *VCAN*, *CLDN9*, *MIOX*, and *TGFBI*) and one oxidative phosphorylation-related gene signature (e.g., *NDUFA4L2*) had a trend of higher expression in tumors compared to adjacent normal tissue in at least 10 cancer types. In contrast, almost all TCA cycle-related gene signatures had lower expression in most cancer types. Additionally, the differential expression levels of glycolysis-related gene signatures were more remarkable compared to oxidative phosphorylation-related gene signatures. This was consistent with a priori knowledge that aerobic glycolysis is a crucial feature in tumorigenesis and tumor development [24–26]. The results also noted significant differences between tumor and normal samples in all 17 cancer types, with three cancer types (CHOL, KICH, and UCEC) showing higher values of fold change. To make the analysis more reliable, we performed differential expression analysis across another nine samples, representing five cancer types, to further validate our findings. Interestingly, we found good agreement between the above two results. For example, *VCAN* was also significantly highly expressed in tumor samples across all cancer types except BLCA (Fig 1C and S1 Fig). These results strongly indicated that dysregulation of respiratory genes was momentous in diverse cancer contexts.

DNA methylation, which acts as a “switch” for gene activation, was one of the first identified and most intensively studied epigenetic regulatory mechanisms [27]. Moreover, the degree of genomic hypomethylation in tumor cells was also closely related to disease progression [28]. Here, we evaluated differential methylation levels of respiratory gene signatures in 16 cancer types with TCGA methylation data (Fig 1E). It was found that most glycolysis-related gene signatures were almost hypomethylated among these cancers. In contrast, many of the 12 oxidative phosphorylation-related gene signatures were hypermethylated across various cancers. Notably, *VCAN* showed remarkable differential methylation in half of the 16 cancer types and was hypomethylated in both renal clear cell carcinoma and renal papillary cell carcinoma. The above results revealed that nearly all respiratory gene signatures were differentially methylated in the promoter region across most cancer types, which further highlights the potential functional contribution of aberrant methylation levels in carcinogenesis [29,30].

To investigate genetic alterations of respiratory gene signatures in cancer, we set out to assess somatic mutation frequency and copy number variation of patients by integrating TCGA data from 33 cancer types. As a result, the overall mutation frequency of respiratory gene signatures was relatively low. Still, the mutation frequency in the tricarboxylic acid cycle stage was comparatively higher than in the other two stages (Fig 1F). More importantly, we also found that *VCAN* was mutated at a much higher frequency in cancer than other gene signatures. This phenomenon suggested that the gene signature we have previously focused on was reliable. Copy number variation (CNV) is an abnormal condition caused by genomic rearrangements and represents a significant genetic alteration in certain malignant tumors [31,32]. It has been proposed that copy number variation might be involved in carcinogenesis through the alterations of gene expression levels in certain cancers [33]. Therefore, we next assessed the CNV patterns of gene signatures involved in the three stages of respiration across different cancer types (Fig 1D). The results showed that *ALDH3B2*, *PKLR*, *SLC2A2*, *GPR87*, and *ATP6V0D2* tended to be widely amplified across cancer types, while *SLC2A5*, *LIPH*, *ATP12A*, and *AGXT* tended to be deleted. Furthermore, 8 of 33 cancer types had few CNV alterations, including KICH, KIRC, KIRP, LAML, PCPG, THCA, and THYM. It was worth noting that the copy number variation of gene signatures in the glycolysis and oxidative phosphorylation stages still exhibited distinct patterns. Moreover, the vast majority of glycolysis-related gene signatures showed extensive CNV amplifications across 25 cancer types.

### Respiratory gene signatures may activate or repress numerous oncogenic pathways among 33 cancer types

**Fig 2:**
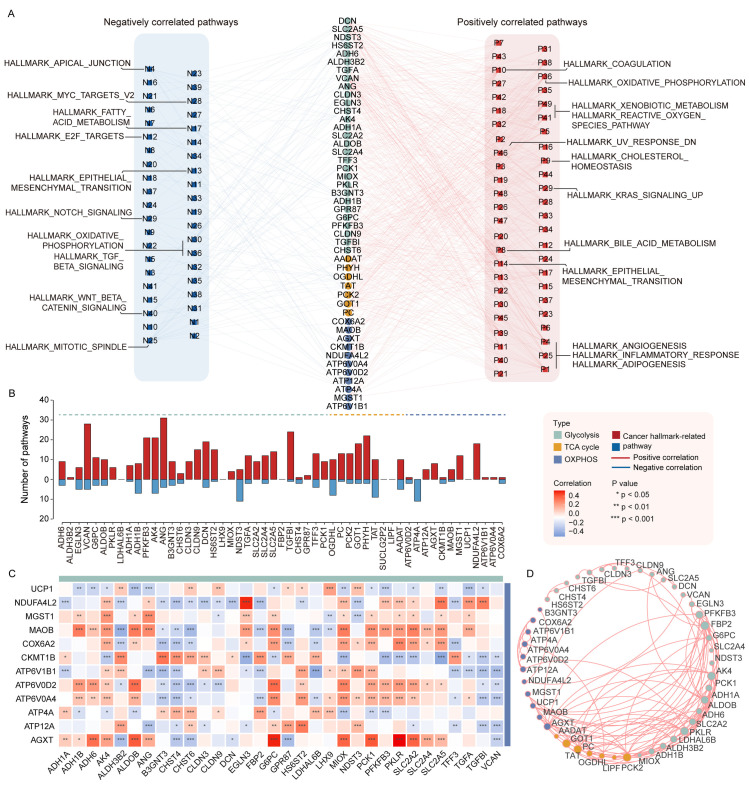
Correlations between respiratory gene signatures and multiple cancer hallmark-related pathways in 33 cancer types. (A) Network diagram indicates the oncogenic pathways positively or negatively correlated with respiratory gene signatures, marked in red and blue, respectively. (B) Bidirectional Histogram shows the number of pathways correlated with each respiratory gene signature. The upper panel in red represents positively correlated pathways, and the bottom panel in blue represents negatively correlated pathways. (C) Heatmap shows Pearson correlation between the expression of glycolysis- and oxidative phosphorylation-related gene signatures. The asterisk character represents the significance of the statistical difference, *p < 0.05; **p < 0.01; ***p < 0.001. (D) The protein-protein interaction network among respiratory gene signatures in the three stages.

In order to further explore the molecular mechanisms of the role of respiratory genes in cancer, we performed Pearson correlation analysis on the expression of respiratory gene signatures and the activity of cancer hallmark-related pathways. We identified a great number of oncogenic pathways that were significantly interlinked with respiratory gene signatures across 33 cancer types (FDR < 0.05, |PCC| > 0.6) (Fig 2A and 2B and S1 Table). Fig 2A illustrated pathways positively and negatively associated with respiratory gene signatures of three different stages. It was observed that almost all glycolysis- and tricarboxylic acid cycle-related gene signatures were dramatically relevant to the activation or inhibition of multiple cancer hallmark-related pathways. In contrast, only a few oxidative phosphorylation-related gene signatures had something to do with oncogenic pathways. Of particular interest, respiratory gene signatures were primarily enriched in several meaningful biological pathways. For instance, angiogenesis, reactive oxygen species, KRAS signaling pathways, and metabolism-related processes like oxidative phosphorylation and bile acid metabolism were positively correlated with the expression of respiratory gene signatures. Notch and Wnt signaling pathways, as well as cell cycle-related processes, such as E2F targets and Myc targets v2, were negatively correlated with respiratory gene signatures. The above discoveries manifested that respiratory genes played an essential role in tumor cell growth, proliferation, and differentiation. Thus, dysregulation of these biological processes might be dominantly responsible for malignant tumorigenesis. This echoed the finding by Xiaoting Hu et al., who reported that the activation of the PI3K-Akt-mTOR pathway might mediate aerobic glycolysis in cancer cells [34]. Therefore, modulating relevant oncogenic pathways, such as the cell cycle, by activating or inhibiting respiratory gene signatures could provide a viable solution for tumor therapy.

Nevertheless, respiratory gene signatures may not exert oncogenic effects in isolation. We assessed whether gene signatures involved in the three stages of respiration were broadly associated with each other. By calculating Pearson correlation coefficients between gene signatures of the three stages, we found that the glycolysis and oxidative phosphorylation stages showed weakly correlated expression patterns compared to the other stages (Figs 2C and S2A and S2B). Intriguingly, we also observed similar results in the corresponding protein-protein interaction network, with fewer interactions between glycolysis and oxidative phosphorylation-related gene signatures (Fig 2D). These results further illustrated the relatively weak link between the glycolytic and oxidative phosphorylation stages in tumor cells. As previously mentioned, most cancer cells exhibited increased glycolysis and generated ATP primarily through this metabolic pathway [9].

### The expression of respiratory gene signatures significantly correlated with survival of patients in multiple cancers

**Fig 3:**
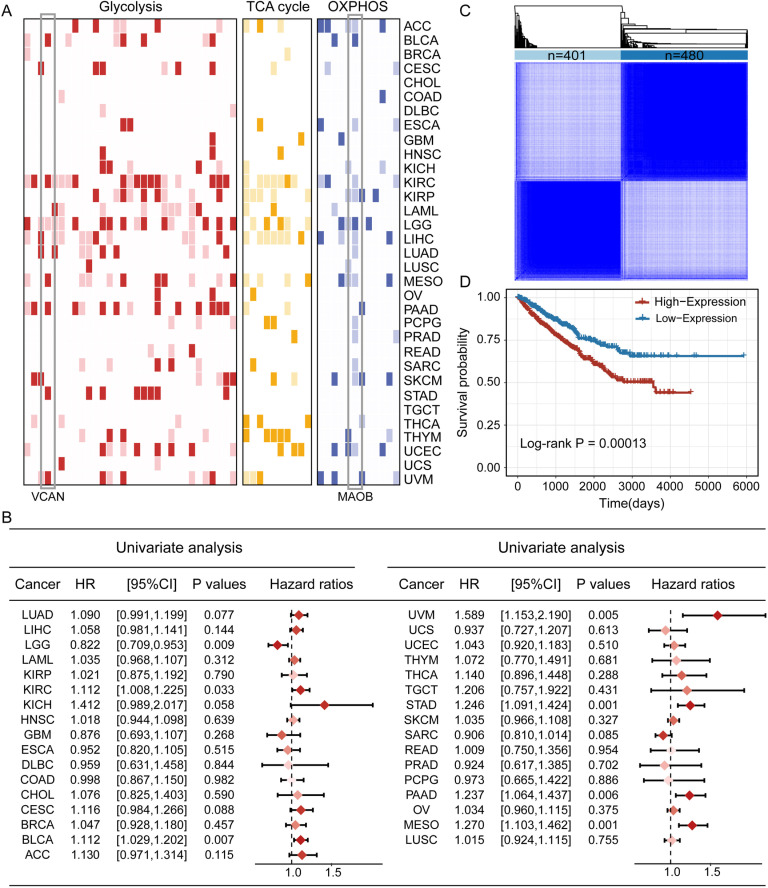
Clinical relevance analysis of respiratory gene signatures in pan-cancer. (A) Heatmap charts the correlation between expression of respiratory gene signatures and patient survival across cancer types. Red, yellow, and blue indicate the gene signatures related to glycolysis, tricarboxylic acid cycle, and oxidative phosphorylation stages, respectively. Dark and light colors indicate risk factors and protective factors, respectively. (B) Univariate cox regression analysis of patients grouped based on the median expression of *VCAN* across 33 cancer types, and darker dots indicate more significant p-values. (C) Consensus clustering for patients with three types of kidney cancer based on the expression of respiratory gene signatures. (D) Kaplan-Meier survival curve of patients grouped by the median expression of respiratory gene signatures in kidney cancer.

In view of the strong association between dysregulation of respiratory gene signatures and cancer development, we intended to assess whether these gene signatures had the potential to predict patient survival. Results showed that several of the 33 cancer types, including ACC, BLCA, KIRC, KIRP, LGG, LIHC, MESO, PAAD, SKCM, THYM, UCEC, and UVM, were significantly associated with the overall survival of patients (Fig 3A). Notably, the expression of multiple respiratory gene signatures was markedly associated with patient prognosis in two other types of kidney cancer except KICH. In addition, we identified two potential oncogenes, one of which is *VCAN*, a gene that had previously received our attention. As shown in Fig 3A, *VCAN* and *TGFBI* were found to be risk factors in six cancer types, and their high expression was associated with worse survival. In contrast, *MAOB* was found to be a protective factor in 12 cancer types, and its high expression was associated with better survival (S3A Fig). These findings confirmed that respiratory gene signatures could indeed predict the overall survival of patients in certain cancer contexts. Taking into account the vital role of *VCAN* in genetic alterations and its oncogenic characteristics in a variety of cancers, we subsequently performed univariate cox regression analysis by dividing patients into high- and low-risk groups based on the median expression of *VCAN* (Fig 3B). Results showed that high expression of *VCAN* was associated with poor prognosis among six cancer types (BLCA, KIRC, MESO, PAAD, STAD, UVM). Moreover, we also conducted univariate cox regression analysis with data from another nine datasets of seven tissues in the GEO database to further validate the oncogenic feature of *VCAN* (S3B Fig). The result reconfirmed that high expression of *VCAN* was associated with poorer survival outcomes in diverse cancers. To sum up, these findings implicated that *VCAN* might function as a vital risk factor during tumorigenesis.

In the end, we observed that many kinds of respiratory gene signatures were remarkably correlated with patient prognosis in KIRC and KIRP, and a few respiratory gene signatures with oncogenic or tumor suppressor features in KICH. Therefore, we meant to further explore whether the expression of respiratory gene signatures would be conducive to the stratification of kidney cancer. Results demonstrated that we identified two subgroups of patients with kidney cancer through consensus clustering on the basis of the global expression levels of respiratory gene signatures (Fig 3C). One subgroup consisted of 401 samples with lower expression of respiratory gene signatures (low-expression group), and the other subgroup of 480 samples with higher expression of respiratory gene signatures (high-expression group). Kaplan-Meier survival curve indicated that patients in the high-expression group had worse survival outcomes compared to those in low-expression group, suggesting that respiratory gene signatures had great potential for prognostic stratification in specific cancer types (Fig 3D).

### Validating the correlation of respiratory gene signatures with biology pathways in kidney cancer

**Fig 4:**
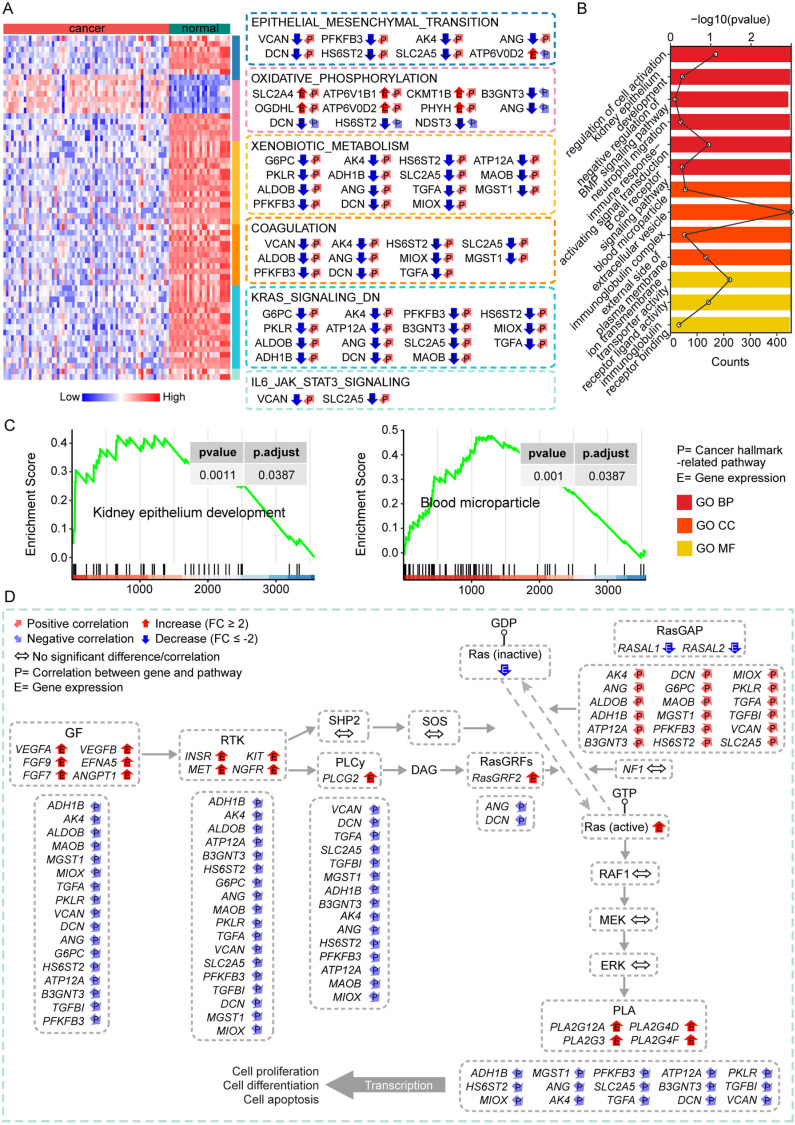
Oncogenic pathways regulated by respiratory gene signatures in KICH. (A) The expression alterations of respiratory gene signatures correlated with several representative pathways in KICH. (B) Bar plot represents the statistical significance of enrichment scores for these representative biological pathways significantly enriched in KICH; line chart represents the number of genes enriched in these pathways. (C) Presentation of GSEA results for two representative pathways. (D) The differential expression of respiratory gene signatures is significantly associated with components of major biological processes in which KRAS is involved in KICH.

To further comprehend the molecular mechanisms by which respiratory gene signatures are involved in cancers, we detailed the expression of respiratory gene signatures remarkably associated with representative cancer hallmark-related pathways in tumor and normal samples in three subclasses of kidney cancer (Figs 4A and S4 and S5A). As a member of the proto-oncogene RAS family, KRAS is involved in the regulation of cell growth, proliferation, and differentiation [35]. It is the most common mutation in the RAS-MAPK signaling pathway and plays a vital role in the pathogenesis of human malignancies [36–38]. The observed correlation between respiratory gene signatures and the KRAS signaling pathway allowed us to show in further detail a schematic representation of the key KRAS-related biochemical processes regulated by respiratory gene signatures. The major pathways involved in KRAS were obtained from the KEGG database. Fig 4D displayed the differential expression patterns of genes involved in the KRAS signaling pathway between tumor and normal tissues in KICH, as well as the broad correlation between these genes and respiratory gene signatures. The results suggested that altered expression patterns of respiratory genes might be a possible factor leading to dysregulation of the signaling pathways they regulate.

Given that the correlation analysis alone could not ascertain our conclusions that respiratory gene signatures might be able to regulate cell proliferation- and metabolism-related processes significantly, we next confirmed previous findings in kidney cancer through gene set enrichment analysis (GSEA). In the first place, we divided all samples in the three types of kidney cancer into high- and low-expression groups based on the median expression of *VCAN*, respectively. Subsequently, we ranked all genes based on their logFC values and performed GSEA. The enrichment results of KICH were visualized in Fig 4B and 4C. It was observed that only biological pathways in the GO database, such as kidney epithelial development, blood microparticle, and BMP signaling pathways, were enriched in KICH. A number of GO and cancer-hallmark pathways, including epithelial mesenchymal transition, KRAS signaling pathways, and metabolism-related pathways like glucose and fatty acid metabolism, were enriched in KIRC (S4B and S4C Fig). Additionally, pathways related to metabolism in GO, KEGG, and Hallmark gene sets, such as bile acid and fatty acid metabolism, blood circulation, reactive oxygen species and KRAS signaling, were enriched in KIRP (S5B and S5C Fig). As expected, the pathways shown to be enriched in the three kidney cancers in the GSEA results were highly consistent with the cancer hallmark-related pathways that we previously identified as significantly regulated via respiratory gene signatures. This strongly confirmed that respiratory genes might play critical roles in tumorigenesis by regulating cell proliferation and metabolism-related processes.

### Identifying respiratory gene signatures that could be potential drug targets

**Fig 5:**
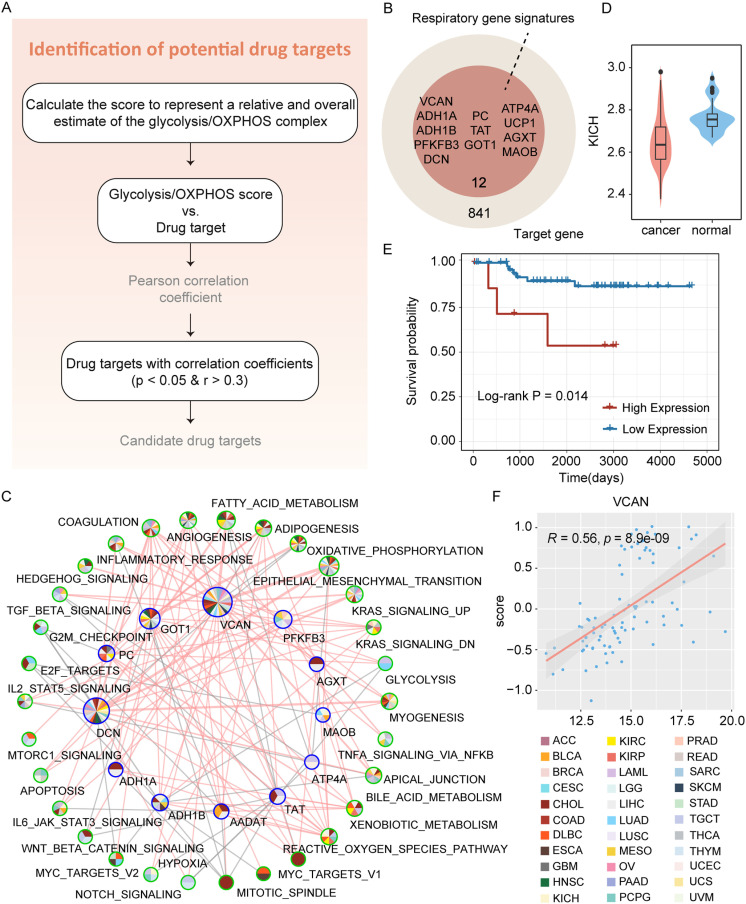
Identification of respiratory gene signatures that could be potential drug targets for patients with impaired respiratory mechanisms. (A) Flowchart for identifying candidate drug targets across various cancer types. (B) Venn diagram charts respiratory gene signatures of three distinct stages that could be potential drug targets. (C) Pan-cancer correlation network between respiratory gene signatures that could be potential drug targets and the cancer hallmark-related pathways. The pie chart colors at each node indicate that drug targets or pathways correlated with that node occurs in cancer types of the corresponding colors. Node size indicates the number of its associated drug targets or pathways. The thickness of the line indicates the number of cancer types in which the two nodes are related. Blue and green borders denote drug targets and pathways, respectively; pink and gray lines denote positive and negative correlations, respectively. (D) Violin Plot represents the expression distribution of *VCAN* in tumor and normal samples of KICH. (E) Kaplan-Meier survival curve represents the prognostic difference in patients grouped by expression values of *VCAN* in KICH. (F) Scatter plot represents Pearson’s correlation between the expression of *VCAN* and glycolysis score in KICH.

The finding that respiratory gene signatures were highly correlated with cell cycle- and metabolism-related pathways may lead us to explore whether these signatures have potential therapeutic implications. However, the macromolecular structure of most human proteins makes it difficult for them to pass through cell membranes and into cells [18]. Therefore, we conducted a two-step analysis and identified a number of respiratory gene signatures in multiple cancer types that could be candidate druggable therapeutic targets (Fig 5A and S2 Table). Since the differences in respiration between normal and tumor cells were mainly manifested in the glycolysis and oxidative phosphorylation stages, we next focused on the results of these two stages in kidney cancer. Above all, the glycolysis score per sample was determined by calculating the mean of normalized expression values of respiratory gene signatures. The oxidative phosphorylation score was also defined similarly, representing the estimated level of overall glycolysis and OXPHOS expression. In the second place, we performed the Pearson correlation analysis between the expression of druggable proteins and glycolysis and OXPHOS scores [22]. As shown in Fig 5B, we finally identified 12 respiratory gene signatures that could be promising therapeutic targets with correlation coefficients more than 0.3 (p < 0.05) in the three subtypes of kidney cancer. At the same time, all of these protein targets were significantly associated with multiple oncogenic pathways across cancer types (Fig 5C). These discoveries further provide promising options for the clinical treatment of tumors. Most importantly, *VCAN* was also a candidate therapeutic target identified in KICH (Fig 5D–5F). This illustrated that abnormal alterations in the expression levels of *VCAN* had significant biomedical implications. Overall, we proposed that *VCAN* might be a candidate target with the potential to treat patients with kidney cancer but required comprehensive experimental and clinical validation in larger sample cohorts.

### Identification of potential small-molecule compounds targeting respiratory gene signatures

**Fig 6:**
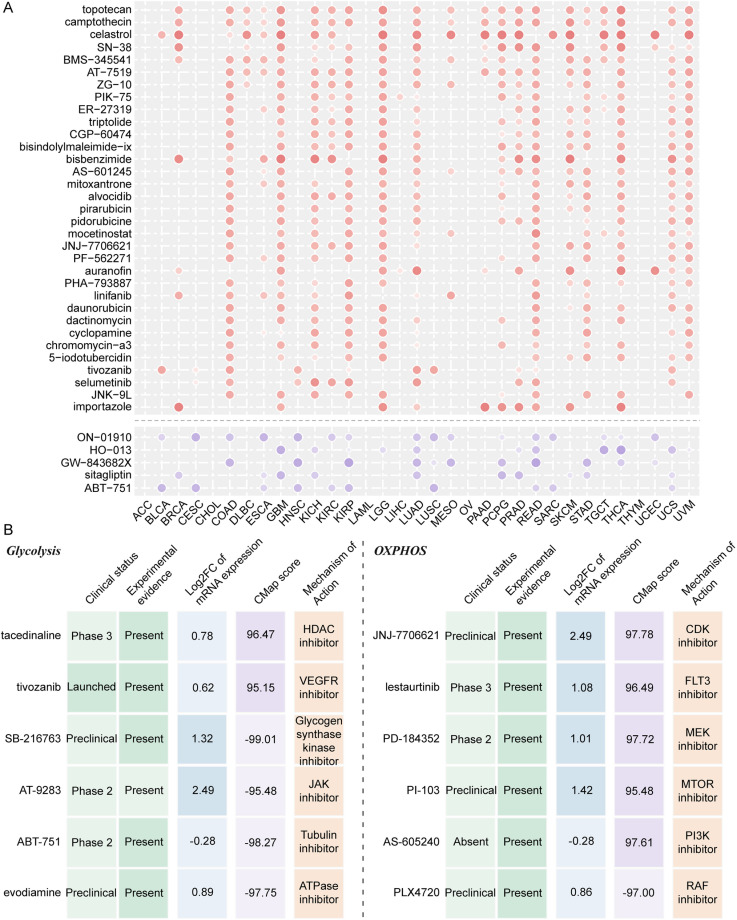
Identification of candidate drugs targeting respiratory gene signatures in pan-cancer. (A) Small-molecule compounds targeting respiratory gene signatures in the oxidative phosphorylation stage. Node size is proportional to the CMap score. Red and purple indicate compounds positively and negatively correlated across cancer types, respectively. (B) Identification of effective therapeutic drugs for patients in KICH according to multi-faceted validation. The left and right panels represent several candidate drugs targeting patients with dysregulated glycolysis and oxidative phosphorylation stages, respectively.

We adopted the Connective Map method to identify candidate small-molecule compounds that might target pathways related to glycolysis and OXPHOS gene signatures or that might regulate their expression. In our study, compounds with CMap scores greater than 95 or less than −95 were considered to have potential therapeutic effects for patients (S3 and S4 Table). By setting a threshold for higher CMap enrichment scores in at least ten cancer types, this analysis yielded 47 and 33 compounds positively associated with glycolysis and oxidative phosphorylation gene signatures, respectively (Figs 6A and S6). Conversely, it also yielded 21 and 5 compounds negatively correlated with glycolysis and oxidative phosphorylation gene signatures, respectively. Nevertheless, the enrichment score alone could not strongly confirm the therapeutic potential of these small-molecule compounds across distinct cancer types. We subsequently conducted an all-around analysis of kidney cancer to assure the reliability of the drug candidates identified through the CMap method (Figs 6B and S7). On the one hand, we calculated the differential expression levels of target genes of drug candidates between normal and tumor tissues. Those with higher fold change values were considered to have greater therapeutic potential for patients. On the other hand, we searched the literature in PubMed to find evidence that candidate compounds exerted therapeutic effects by targeting pathways related to respiratory gene signatures or modulating their expression.

Collectively, multiple protein synthesis inhibitors were found to be positively and negatively connected with respiratory gene signatures in the glycolysis and oxidative phosphorylation stages across three renal cancers, including CDK, HDAC, MEK, PI3K, PLK, and mTOR. This indicated that targeting these candidate small-molecule compounds might result in altered expression of respiratory gene signatures, thereby exerting specific therapeutic effects by modulating the oncogenic pathways they regulated. What’s more important is that experimental and clinical evidence for these candidate compounds targeting respiratory gene signatures could be found in PubMed [39–43]. Our findings showed that respiratory genes indeed played a critical role in cancer therapy through a series of cellular physiological activities. For example, they might influence tumor cell growth, proliferation, and differentiation through the activation or inhibition of Notch, MAPK, PI3K, and NF-KB signaling pathways [44]. To a large extent, these cell cycle-related pathways were consistent with those identified in our prior studies that were notably regulated by respiratory gene signatures. Therefore, we had robust clinical and computational evidence indicating that these small-molecule compounds targeting glycolysis and oxidative phosphorylation hold promising therapeutic potential.

## Discussion

Normal cells mainly rely on mitochondrial oxidative phosphorylation to generate energy and perform glycolysis under hypoxic conditions. Contrary to general trends, tumor cells grow faster and preferentially utilize glycolysis (glucose-pyruvate-lactate) to produce ATP rapidly, even in the presence of large amounts of oxygen [45,46]. This is a unique metabolic feature of tumor cells that can promote their growth and proliferation, which in turn leads to cancer. Therefore, glycolytic reprogramming is a major characteristic of cancer and plays a crucial role in cancer progression. While previous research has indicated that respiratory processes may be involved in carcinogenesis, a comprehensive analysis of all three stages of cellular respiration across pan-cancer has not been conducted. Our multidimensional study could better dissect the molecular mechanisms of respiratory gene dysregulation in different cancer types. In our study, we obtained respiratory genes of three stages from databases and screened a total of 53 gene signatures via differential expression analysis. We systematically profiled these gene signatures to elucidate their importance in various cancers, including characterization of genetic alterations, association of biological pathways, co-expression networks, prognostic potential, and identification of targeted drugs.

Evidence has accrued that respiratory genes may be involved in carcinogenesis [47]. We specifically analyzed the expression perturbations and molecular alterations of respiratory genes among different cancer types. Differential expression results indicated that the expression of almost all respiratory gene signatures was markedly different between tumor and normal samples of multiple cancers. In particular, *VCAN* was highly expressed in 11 out of 17 cancers. We also observed widespread alterations in the mutational landscape. Notably, the frequency of mutations in *VCAN* was significantly higher than in other gene signatures, further highlighting its oncogenic potential. Methylation analysis revealed widespread differential methylation of respiratory gene signatures in all cancer types except KIRC, KIRP, KICH, LAML, LGG, and UVM. In addition, respiratory gene signatures exhibited altered copy number patterns. For example, *VCAN* showed CNV amplification in six cancers and CNV deletion in two. These findings suggested that there might be some functional links between the dysregulation of respiratory genes and tumorigenesis.

The occurrence and development of cancer is a complex regulatory network. We identified cancer-hallmark pathways regulated by respiratory gene signatures across 33 cancer types through Pearson correlation analysis. Most of these pathways were associated with angiogenesis, metabolism and cell proliferation, such as KRAS signaling pathway. This meant that respiratory genes might contribute to cellular carcinogenesis by affecting these biological processes. There is evidence that lipopolysaccharide (LPS) might mediate aerobic glycolysis through the activation of the PI3K-Akt-mTOR/PFKFB3 pathway during LPS-induced sepsis [34]. Furthermore, cancer cells generate ATP through glycolysis and the TCA cycle associated with oxidative phosphorylation [48]. We constructed a co-expression network to investigate whether the three stages of respiratory processes function independently in the context of cancer. Results showed more interaction between the glycolysis and TCA cycle stages and less interaction with the oxidative phosphorylation stage. This was consistent with a priori knowledge that most tumor cells depend largely on glycolysis to meet their energy requirements [49]. Subsequently, survival analysis has also revealed the oncogenic or tumor suppressor potential of many respiratory gene signatures. In particular, high expression of *VCAN* was associated with poor prognosis of patients in a variety of cancers. Moreover, we found that multiple respiratory gene signatures were remarkably correlated with patient prognosis in three types of kidney cancer. Consensus clustering analysis indicated that the expression of respiratory genes may contribute to the stratification of kidney cancer patients. Therefore, we focused primarily on KICH, KIRC, and KIRP in the subsequent analysis.

**Fig 7:**
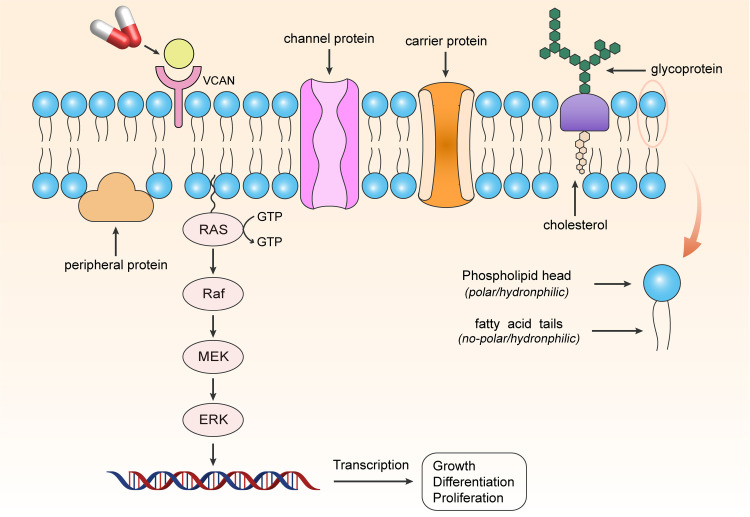
Schematic representation of small-molecule compounds acting on a therapeutic target, *VCAN*, to regulate tumor cell growth, differentiation, and proliferation via the MAPK signaling pathway.

Targeted therapy has brought new opportunities for the clinical treatment of various cancers [50]. Cellular respiration plays a crucial role in carcinogenesis, especially glycolysis, which is the backbone of cancer cell metabolism. The TCA cycle is recognized as a central pathway for cellular energy production and precursors for biosynthetic pathways. Oxidative phosphorylation, the final step of cellular respiration, has been linked to cancer progression. Therefore, key factors regulated by respiratory gene signatures in signaling pathways could become appealing targets for anti-cancer drugs. The stratified treatment strategy we proposed holds great biomedical implications for future clinical applications. Specifically, our study identified many respiratory gene signatures that could be potential therapeutic targets and small-molecule compounds targeting signaling pathways related to respiratory gene signatures. Taking Fig 7 as an example, the targeted drug for anti-cancer therapy acted on *VCAN*,  which we had previously identified as a drug target, and further regulated tumor cell growth, differentiation, and proliferation by affecting the signal transduction pathway it mediates (e.g., MAPK signaling pathway), thereby exerting a therapeutic effect. Unfortunately, we still face the problem that the specific way *VCAN* participates in regulation is still unclear: whether it is transmembrane or acting on the cell membrane, which still needs to be thoroughly investigated in the future.

Taken together, our study untangled the full range of molecular alterations of respiratory genes in pan-cancer to assess their underlying molecular mechanisms in tumorigenesis and developed promising therapeutic strategies. This analysis has not only laid the groundwork for deciphering the dysregulation of respiratory processes in tumor cells but also provided valuable clues to the development of relevant drug targets.

## Supporting information

S1 Fig Box plot charts the expression distribution of VCAN across normal and tumor tissues in seven GEO samples. (TIF)

S2 Fig Correlation among the expression of respiratory gene signatures. **(A)** Heatmap shows Pearson correlation between the expression of glycolysis and the TCA cycle-related gene signatures. **(B)** Heatmap shows Pearson correlation between the expression of the TCA cycle and oxidative phosphorylation-related gene signatures. The asterisk character represents the significance of the statistical difference, *p < 0.05; **p < 0.01; ***p < 0.001.
(TIF)

S3 Fig Prognostic capacity of MAOB and VCAN. **(A)** Kaplan-Meier survival curves of patients grouped based on the expression of tumor suppressor gene *MAOB* in TCGA data. **(B)** Univariate cox regression analysis of patients grouped based on the median expression of *VCAN* across nine GEO samples, and darker dots indicate more significant p-values.
(TIF)

S4 Fig Oncogenic pathways regulated by respiratory gene signatures in KIRC. (TIF)

S5 Fig Oncogenic pathways regulated by respiratory gene signatures in KIRP. (TIF)

S6 Fig Small-molecule compounds targeting respiratory gene signatures in the glycolysis stage. Node size is proportional to the CMap score. Red and purple indicate compounds positively and negatively correlated across cancer types, respectively.
(TIF)

S7 Fig Identification of effective therapeutic drugs for patients with kidney cancer according to multi-faceted validation. **(A-B)** Several representative candidate small-molecule compounds identified in KIRC and KIRP, respectively.
(TIF)

S1 Table The correlation of respiratory gene signatures and cancer hallmark-related pathways. (XLSX)

S2 Table Respiratory gene signatures identified as potential drug targets across cancer types. (XLSX)

S3 Table The identification of drugs targeting respiratory gene signatures involved in glycolysis stage via the CMap method. (XLSX)

S4 Table The identification of drugs targeting respiratory gene signatures involved in the oxidative phosphorylation stage via the CMap method. (XLSX)
