# Supplementary figures and images for: Comprehensive characterization of respiratory genes based on a computational framework in pan-cancer to develop stratified treatment strategies
Source: PLoS Comput Biol. 2025 Apr 9;21(4):e1012963. doi: 10.1371/journal.pcbi.1012963 (PMC11981224; doi:10.1371/journal.pcbi.1012963)

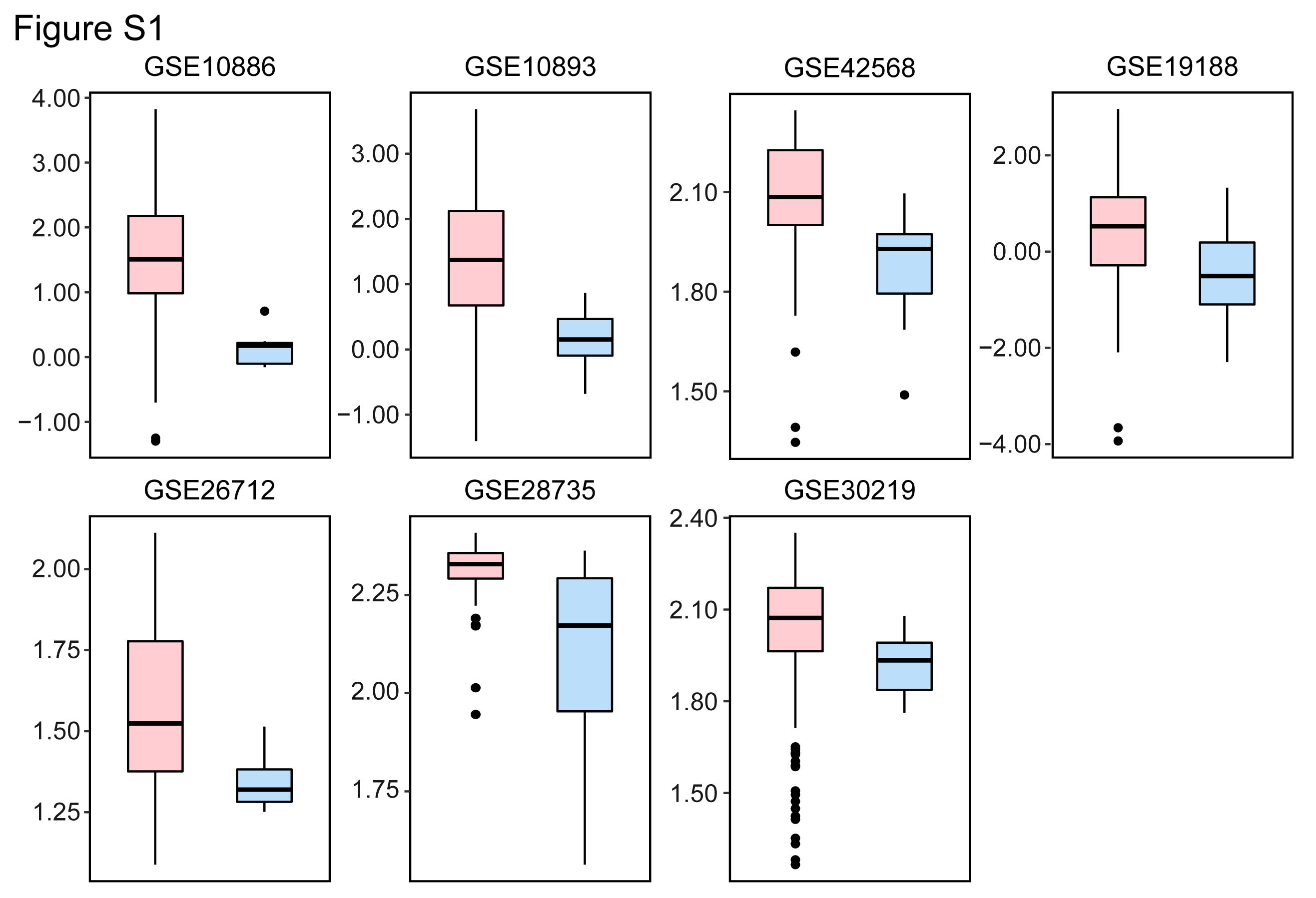

Supplement: S1 Fig — (TIF) [file pcbi.1012963.s001.tif]

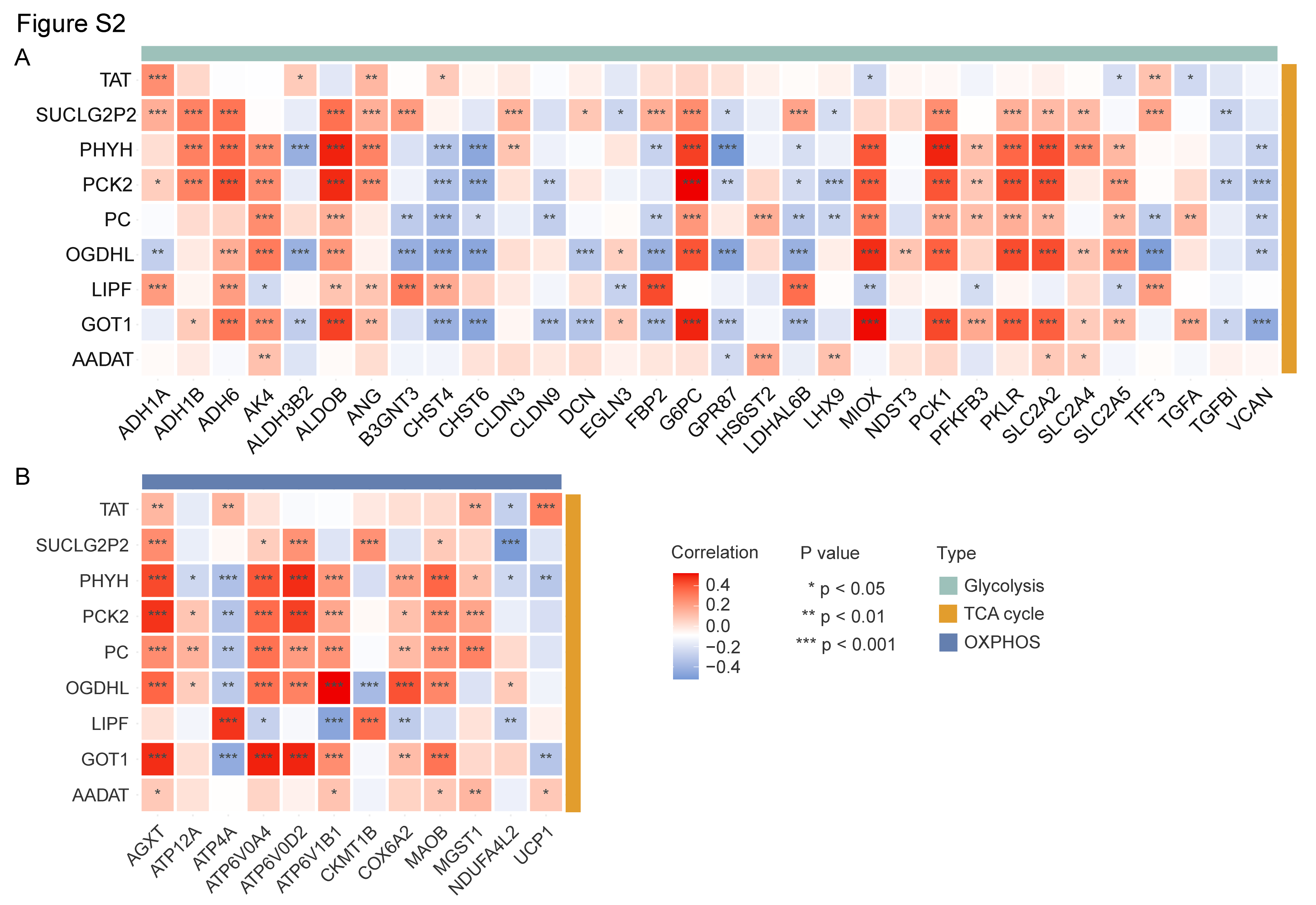

Supplement: S2 Fig — (A) Heatmap shows Pearson correlation between the expression of glycolysis and the TCA cycle-related gene signatures. (B) Heatmap shows Pearson correlation between the expression of the TCA cycle and oxidative phosphorylation-related gene signatures. The asterisk character represents the significance of the statistical difference, *p < 0.05; **p < 0.01; ***p < 0.001. (TIF) [file pcbi.1012963.s002.tif]

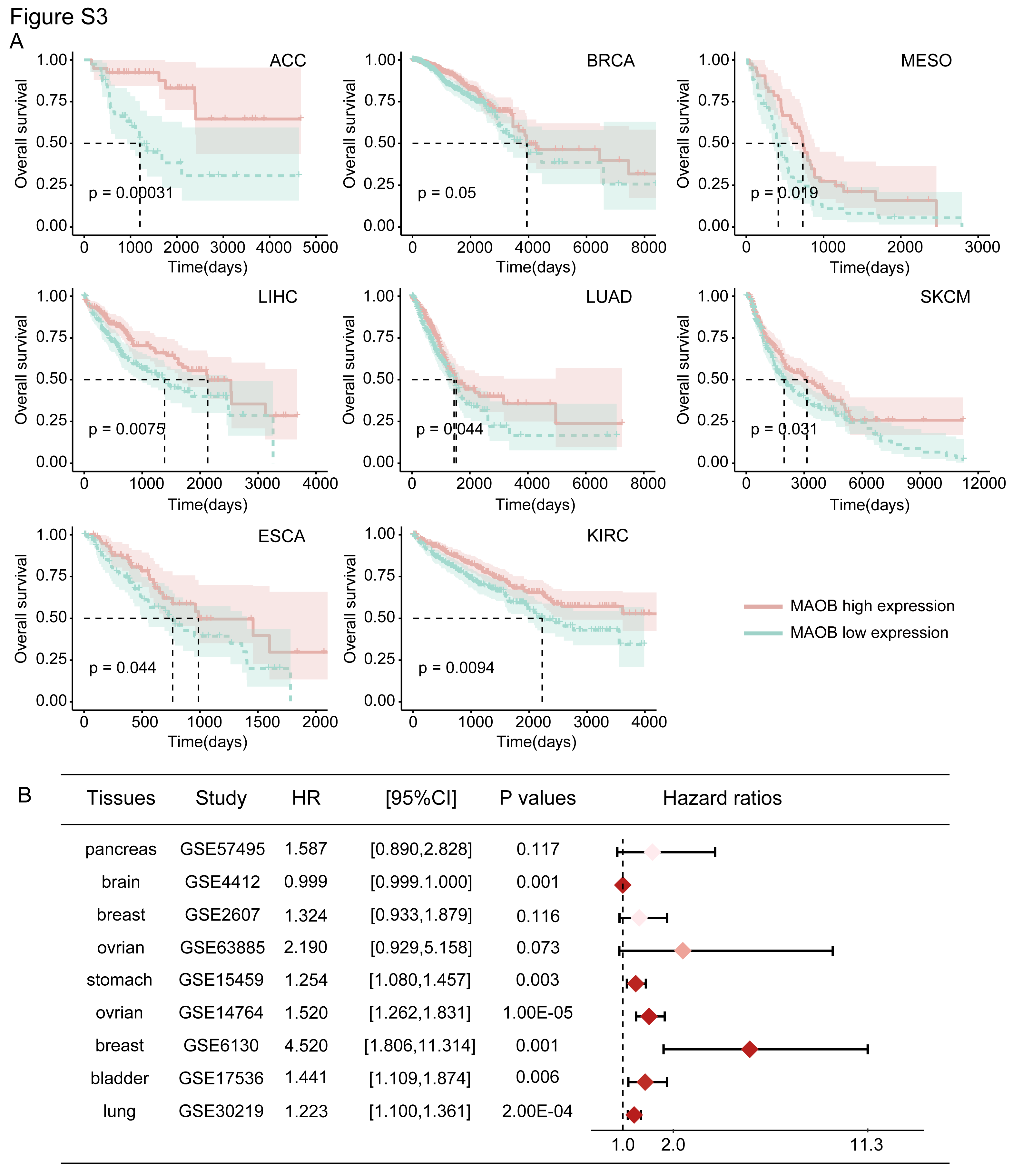

Supplement: S3 Fig — (A) Kaplan-Meier survival curves of patients grouped based on the expression of tumor suppressor gene MAOB in TCGA data. (B) Univariate cox regression analysis of patients grouped based on the median expression of VCAN across nine GEO samples, and darker dots indicate more significant p-values. (TIF) [file pcbi.1012963.s003.tif]

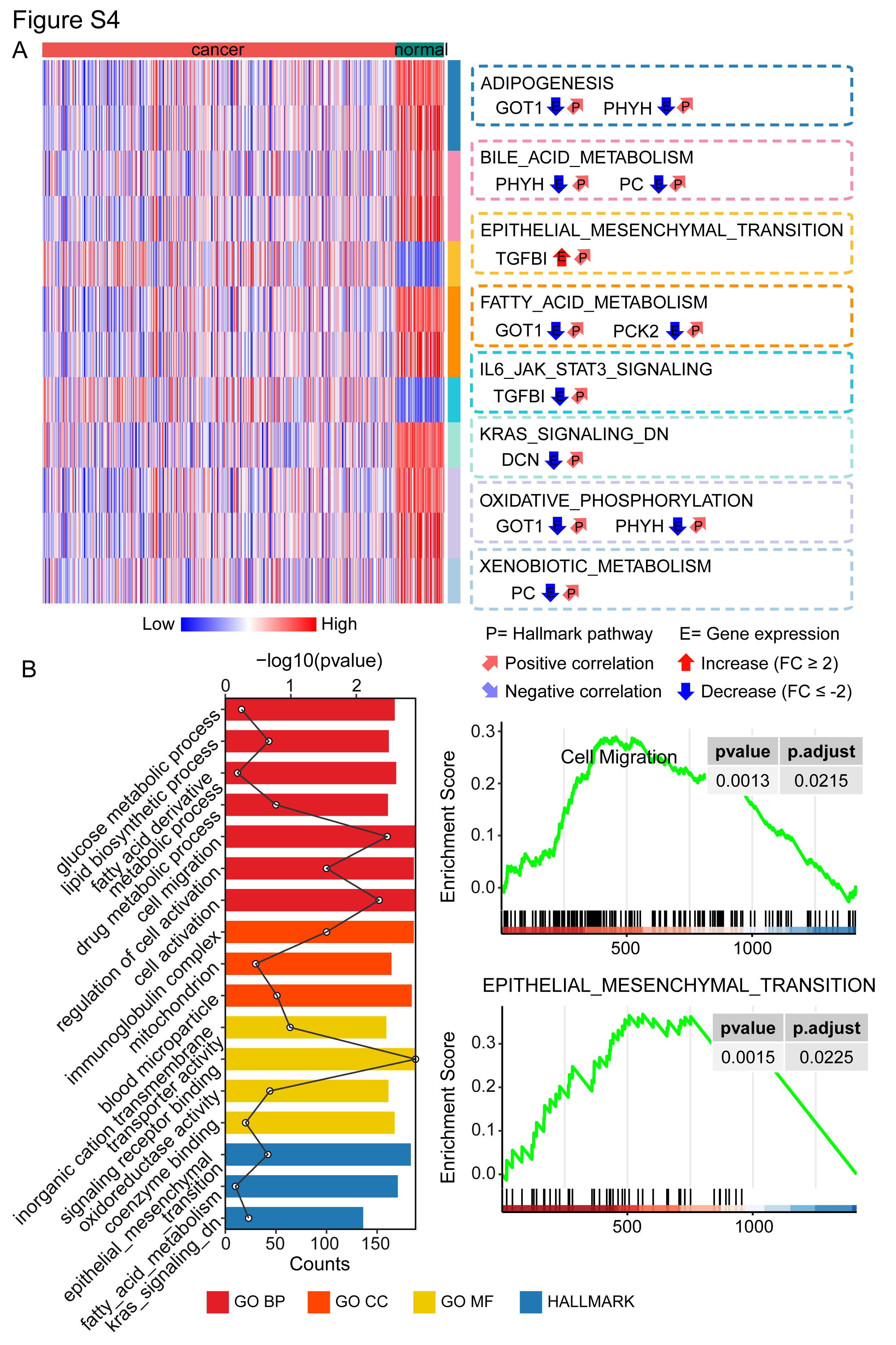

Supplement: S4 Fig — (TIF) [file pcbi.1012963.s004.tif]

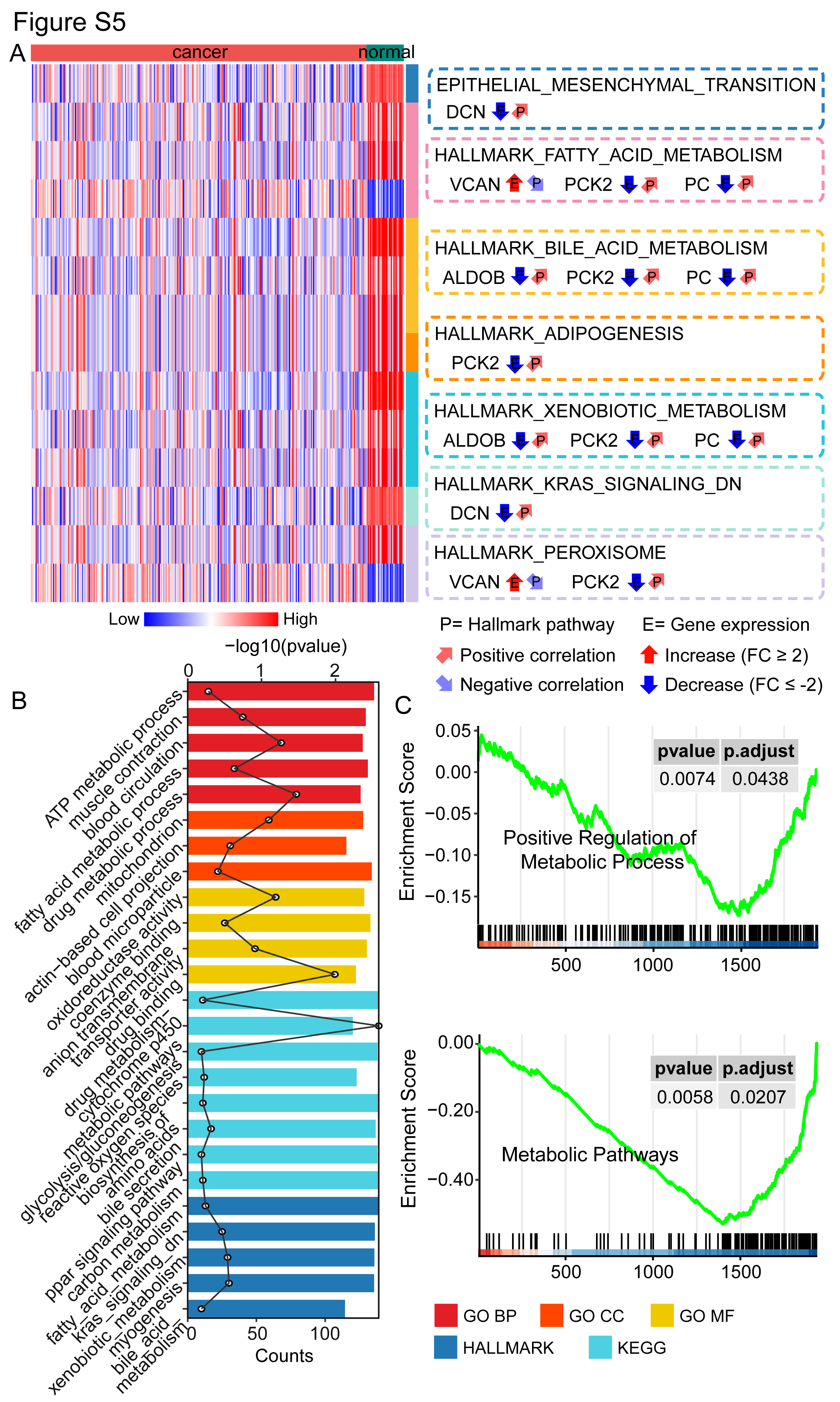

Supplement: S5 Fig — (TIF) [file pcbi.1012963.s005.tif]

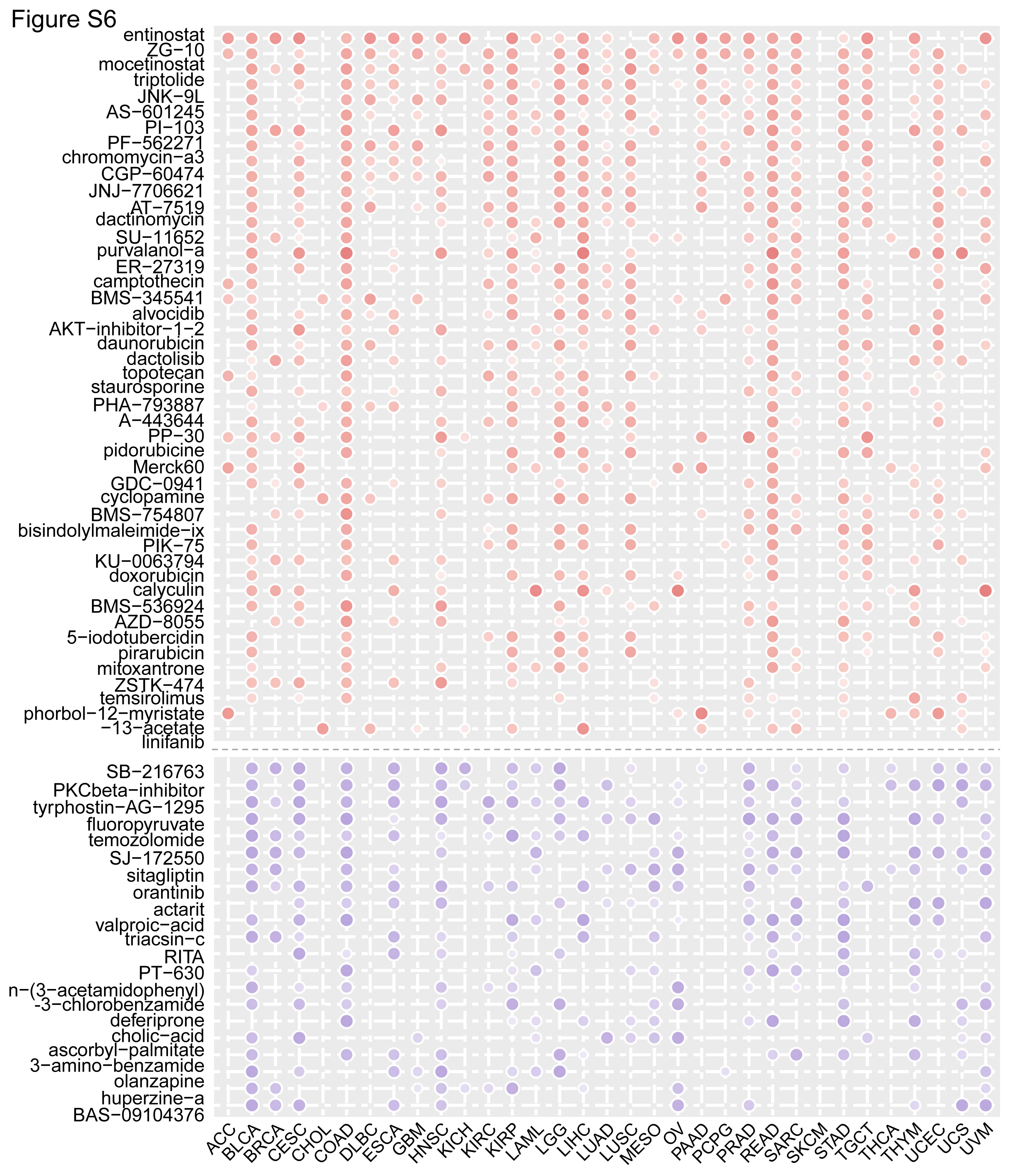

Supplement: S6 Fig — Node size is proportional to the CMap score. Red and purple indicate compounds positively and negatively correlated across cancer types, respectively. (TIF) [file pcbi.1012963.s006.tif]

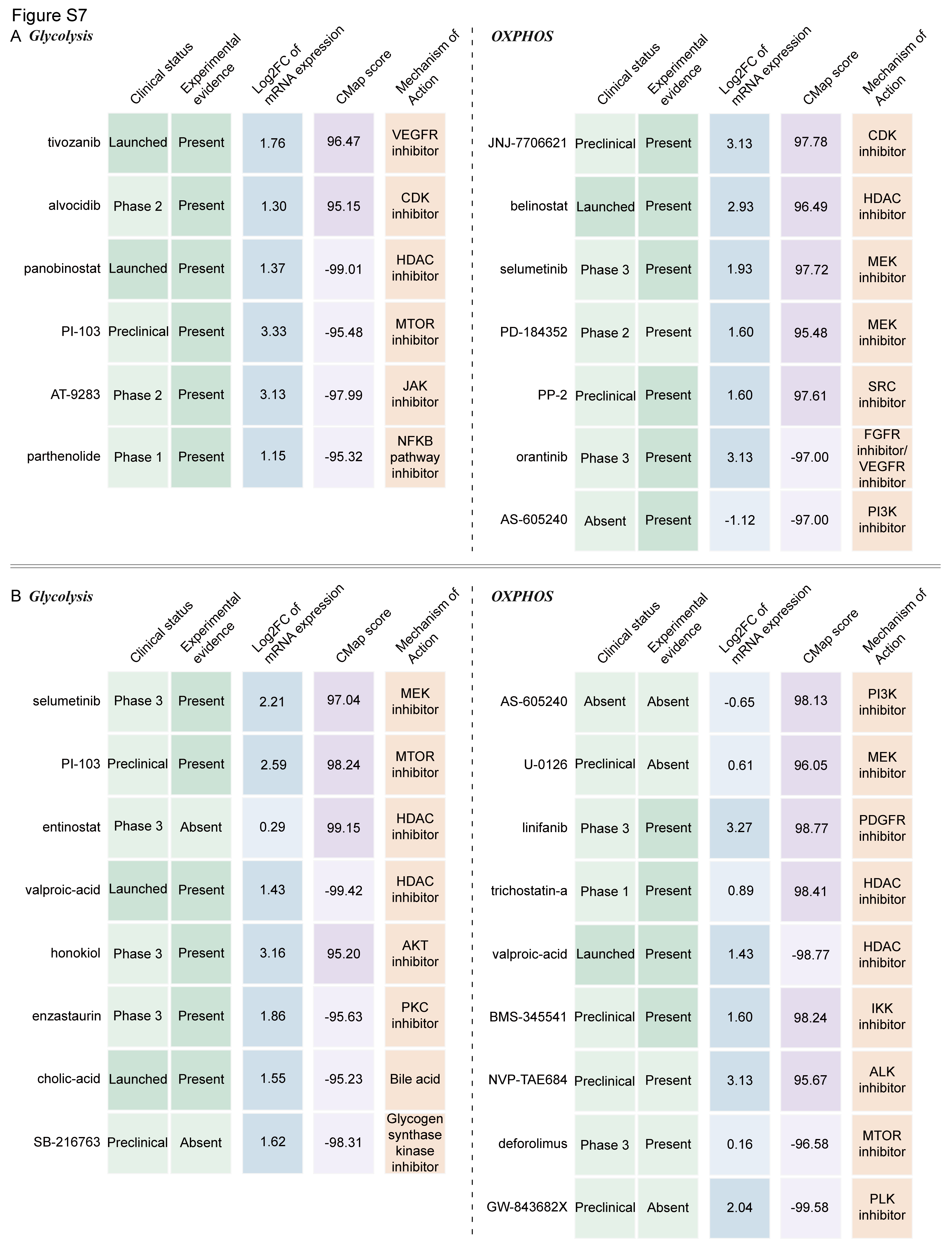

Supplement: S7 Fig — (A-B) Several representative candidate small-molecule compounds identified in KIRC and KIRP, respectively. (TIF) [file pcbi.1012963.s007.tif]
